# Challenges in the size analysis of a silica nanoparticle mixture as candidate certified reference material

**DOI:** 10.1007/s11051-016-3474-2

**Published:** 2016-06-23

**Authors:** Vikram Kestens, Gert Roebben, Jan Herrmann, Åsa Jämting, Victoria Coleman, Caterina Minelli, Charles Clifford, Pieter-Jan De Temmerman, Jan Mast, Liu Junjie, Frank Babick, Helmut Cölfen, Hendrik Emons

**Affiliations:** Institute for Reference Materials and Measurements (IRMM), Joint Research Centre (JRC), European Commission, Retieseweg 111, 2440 Geel, Belgium; National Measurement Institute Australia, Nanometrology Section, 36 Bradfield Road, West Lindfield, NSW 2070 Australia; Analytical Science Division, National Physical Laboratory, Hampton Road, Teddington, Middlesex, TW11 0LW UK; Service Electron Microscopy, Veterinary and Agrochemical Research Centre (CODA-CERVA), Groeselenberg 99, 1180 Brussels, Belgium; Division of Nanoscale Measurement and Advanced Materials, National Institute of Metrology, No. 18, Bei San Huan Dong Lu, Beijing, China; Institut für Verfahrens- und Umwelttechnik, Technische Universität Dresden, 01062 Dresden, Germany; Physical Chemistry, Department of Chemistry, University of Konstanz, Universitätsstraße 10, 78457 Constance, Germany

**Keywords:** Certified reference material, Interlaboratory comparison study, Measurement uncertainty, Measurand, Particle size analysis, Quality assurance, Silica nanoparticles, Nanotechnology

## Abstract

A new certified reference material for quality control of nanoparticle size analysis methods has been developed and produced by the Institute for Reference Materials and Measurements of the European Commission’s Joint Research Centre. The material, ERM-FD102, consists of an aqueous suspension of a mixture of silica nanoparticle populations of distinct particle size and origin. The characterisation relied on an interlaboratory comparison study in which 30 laboratories of demonstrated competence participated with a variety of techniques for particle size analysis. After scrutinising the received datasets, certified and indicative values for different method-defined equivalent diameters that are specific for dynamic light scattering (DLS), centrifugal liquid sedimentation (CLS), scanning and transmission electron microscopy (SEM and TEM), atomic force microscopy (AFM), particle tracking analysis (PTA) and asymmetrical-flow field-flow fractionation (AF4) were assigned. The value assignment was a particular challenge because metrological concepts were not always interpreted uniformly across all participating laboratories. This paper presents the main elements and results of the ERM-FD102 characterisation study and discusses in particular the key issues of measurand definition and the estimation of measurement uncertainty.

**Electronic supplementary material:**

The online version of this article (doi:10.1007/s11051-016-3474-2) contains supplementary material, which is available to authorised users.

## Introduction

The European Commission (EC) adopted a Recommendation ‘on the definition of nanomaterial’ (EC 2011) in October 2011. This Recommendation (2011/696/EU) defines a nanomaterial in general as a “*natural, incidental or manufactured material containing particles in an unbound state or as an aggregate or as an agglomerate and where, for50 %or more of the particles in the number size distribution, one or more external dimensions is in the size range of 1 nm*–*100* *nm*”. An implementation of the recommendation requires reliable measurement techniques that can, preferably under routine conditions and with high-throughput, accurately measure the size of nano-objects (e.g. nanoparticles) in the defined particle size range around the number size distribution threshold of 50 % (Linsinger et al. 2012; De Temmerman et al. 2014a). Because test results obtained with such measurement methods may be used, for example, for product registration and labelling purposes, their quality and reliability must be assured through the application of fully validated analytical methods.

The basis of method validation is usually a well-designed experimental study during which reference materials are analysed to evaluate method performance characteristics such as selectivity, sensitivity, limit of detection, limit of quantification, precision and trueness (Eurachem 1998). The latter, which reflects the closeness of agreement between the average of an infinite number of replicate measured quantity values and a reference quantity value, is quantitatively expressed in terms of bias (JCGM 2012). One of the accepted principles for assessing the measurement bias is by analysing a fit-for-purpose material that is sufficiently homogeneous and stable and that comes with an accepted reference value. Certified reference materials (CRMs), as described in ISO Guide 30 (2015a), meet these quality criteria.

Over the last decade, different CRMs have been produced for various kinds of calibration and laboratory quality assurance purposes such as control charts and validation of particle size analysis (PSA) methods (NIST 2007; Braun et al. 2011a, b; Linsinger et al. 2011; Franks et al. 2012; De Temmerman 2014b). However, the majority of the available (nano)particle CRMs have a monomodal and relatively narrow particle size distribution (PSD) which makes them less suitable for validating methods that are intended for PSA of polydisperse particulate materials. As a consequence, new CRMs with a more polydisperse size distribution that extends both in the nano- and in the submicrometre-scale range are required to validate methods in the context of the EC nanomaterial definition. As a first step in this direction, the Institute for Reference Materials and Measurements (IRMM) of the Joint Research Centre (JRC) of the EC has developed and produced the first CRM (ERM-FD102) that consists of a mixture of industrially sourced near-spherical silica nanoparticles with an essentially bimodal PSD (Kestens and Roebben 2014).

The production process of ERM-FD102 was performed according to the requirements prescribed in ISO Guide 34 (2009). Equivalence between the 2061 sample units produced was guaranteed by the results of a homogeneity study. Short- and long-term stability studies were used to determine suitable transport and storage conditions. The focus of this paper is on the part of the CRM production process where a number of certified and indicative values were assigned on the basis of a characterisation^1^ study which comprised worldwide interlaboratory comparisons (ILCs) of expert laboratories with demonstrated competence. In the characterisation study of ERM-FD102, a clear choice was made to certify several values, each corresponding with a different method-defined (sometimes also referred to as operationally defined) property or measurand. The metrological concept of *measurand* is defined by the international vocabulary of metrology (VIM) as the *quantity intended to be measured* (JCGM 2012). PSA methods *intend* to determine PSDs or characteristic particle diameters (or radii) of particulate matter. Following this definition, one may consider *particle diameter* as being the unambiguous and logical measurand.

This paper elaborates some of the main metrological challenges that were encountered during the characterisation study: measurand definition and its minimum required specifications to allow comparability of results and the estimation of measurement uncertainties.

## Materials and methods

### Candidate CRM

The candidate CRM is an aqueous suspension of a mixture of near-spherical silica nanoparticles with distinct sizes. Two commercially available colloidal silica polishing suspensions, Köstrosol 1530 (Chemiewerk Bad Köstritz GmbH, Bad Köstritz, DE) and Klebosol 30R50 (AZ Electronic Materials, Trosly Breuil, FR) were selected as starting materials. Köstrosol 1530 provided nanoparticles with a diameter of nominally 20 nm and Klebosol 30R50 was the source material of nanoparticles of nominally 80 nm in diameter. The latter also contained a fraction of nanoparticles with a diameter of nominally 40 nm (about 1:2 number ratio of 40 and 80 nm particles). An overview of relevant information on physical properties of the two starting materials, as provided by the product manufacturers and additionally obtained by preliminary TEM measurements, is given in Supplemental Table S1. The Köstrosol 1530 and Klebosol 30R50 starting materials (each with a particle mass fraction of 300 g/kg) were first diluted individually in high-quality reverse osmosis purified water to a nominal particle mass concentration of 10 g/kg and 2.5 g/kg, respectively. Five volume parts of the diluted Köstrosol 1530 sub-batch were mixed with one volume part of the diluted Klebosol 30R50 sub-batch. A total of 2061 glass ampoules of 10 mL were semi-automatically filled with approximately 9 mL of suspension, flame sealed and labelled with a unique sample unit identification number that reflects the ampouling sequence.

The 21:1 particle mass (or 320:1 particle number) ratio of 20 nm particles to 80 nm particles was chosen based on preliminary investigations which revealed that most of the techniques that were targeted for the ILCs would be able to (simultaneously) measure the particle size of the two dominating particle populations at this ratio. Throughout the paper, the particles with nominal diameter values of 20 nm and 80 nm will be further referred to as particles belonging to size class A and size class B, respectively. The fraction of particles with a nominal diameter of 40 nm was not considered in the study and during measurements.

Homogeneity and stability studies were specifically designed to determine quantitatively the degree of inhomogeneity between and within the processed sample units, and to determine appropriate shipping (short-term stability) and storage (long-term stability) conditions. The relative standard uncertainties (*u*_bb_) for the between-unit homogeneity of size class A, determined by DLS and CLS, were 3.2 % and 1.2 %, respectively. Similarly, uncertainties of 0.6 % (DLS) and 0.4 % (CLS) were estimated for size class B. The uncertainty contributions *u*_sts_ that were estimated according to an approach first described by Lamberty et al. (1998) from the short-term stability study data, and which account for 1 week at 60 °C, were 0.4 % (DLS) and 0.1 % (CLS) for size class A and 0.2 % (DLS) and 0.1 % (CLS) for size class B. The uncertainty of the particle size values for a shelf life of 24 months at (18 ± 5) °C was calculated according to the procedures described by Linsinger et al. (2001). For size class A, these relative standard uncertainties (*u*_lts_) were 2.6 % (DLS) and 1.1 % (CLS). For size class B, *u*_lts_ was 0.8 % (DLS) and 0.1 % (CLS). The estimated uncertainty components *u*_bb_, *u*_sts_ and *u*_lts_ were used in the overall uncertainty budgets of the certified and indicative values for the different methods.

Full details of the homogeneity and stability study setups and results are available in the certification report of ERM-FD102 (Kestens and Roebben 2014).

### Characterisation study

**Fig. 1:**
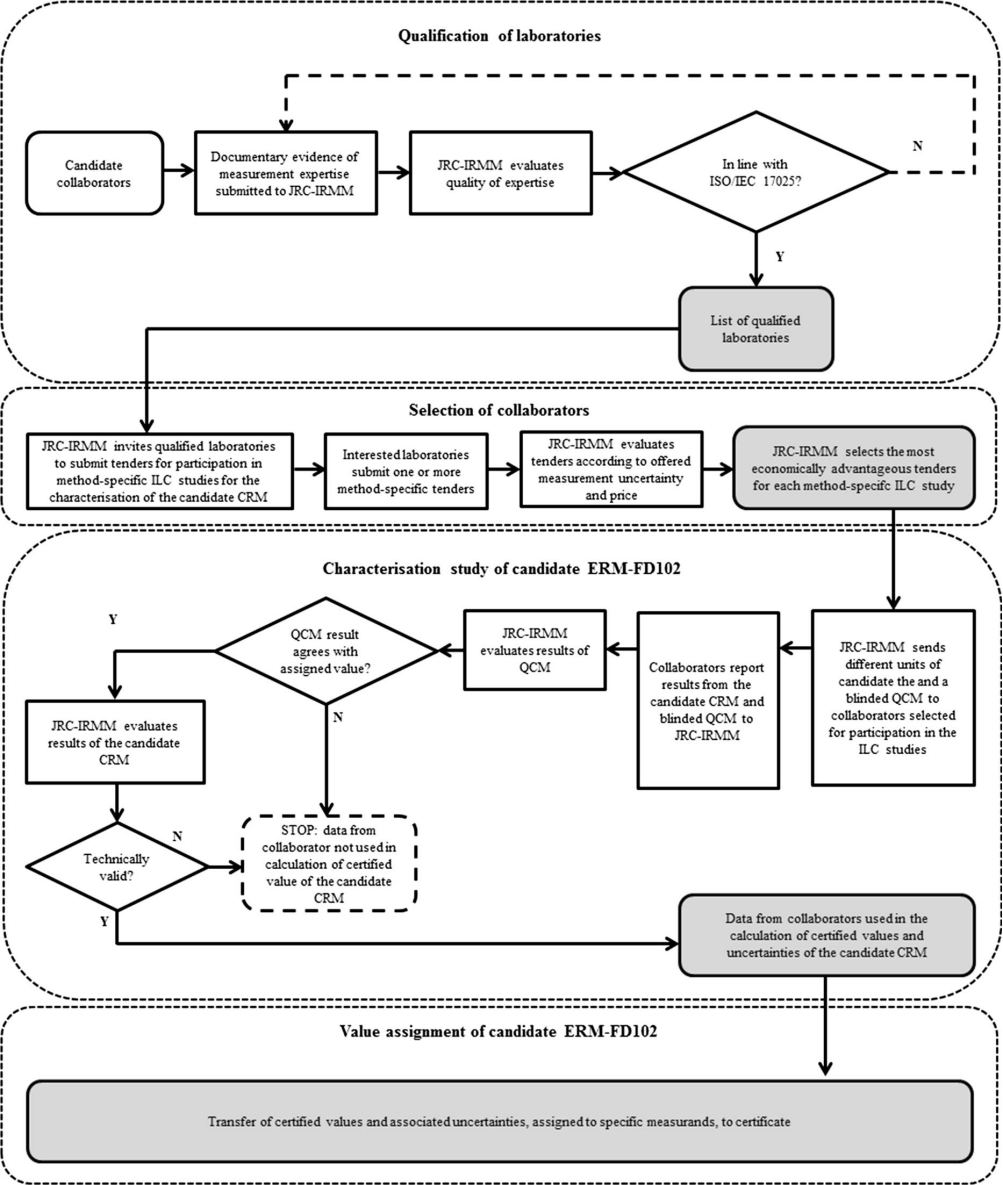
Flowchart showing the qualification and selection process of candidate collaborators and the successive steps of the characterisation study (through the organisation of method-specific ILC studies) which led to the calculation and assignment of different certified values and uncertainties

ISO Guide 35 (2006) recommends for method-defined properties that certified values are determined through an ILC study between qualified expert laboratories. The entire process, which starts with the qualification and selection of the collaborating laboratories and leads to the assignment of certified values using the technically valid ILC data from the characterisation study, can be generally presented as follows (Fig. 1).

JRC-IRMM qualifies candidate laboratories for participation in reference material (RM) characterisation studies based on documented evidence of the laboratory expertise in the specifically required measurement field, e.g. based on ISO/IEC 17025 accreditation. However, only a few laboratories have relevant PSA methods in the scope of their accreditation. To better populate an existing list of qualified collaborators, JRC-IRMM organised a proficiency test (PT) for particle size (and zeta potential) measurements on monomodal aqueous suspensions of silica nanoparticles in 2010 (Lamberty et al. 2011). The performance of each laboratory that participated in the PT scheme, as well as the measurement capability of other laboratories that were unable to participate in the 2010 PT scheme but which were interested to participate in the ERM-FD102 ILC study, was evaluated with respect to the quality of reporting and measurement competence according to the requirements of ISO/IEC 17025 (2005) and ISO/IEC 17043 (2010), respectively.

From the established list of qualified collaborators, 60 laboratories were invited to submit a tender based on a provided detailed measurement protocol. The technically valid tenders were scored and ranked according to pre-defined criteria such as price and offered measurement uncertainty. Finally, 31 of the qualified laboratories received three units of the candidate CRM (labelled as ERM-FD102) and, depending on the method, one or more units of a quality control material (QCM) and a measurement protocol. The protocols included a measurement scheme (i.e. the three units had to be analysed on different days and multiple replicates per unit per day were required) as well as instructions for sample handling and relevant measurement method parameters. A summary of the different measurement conditions as provided to the laboratories via the measurement protocol is given in Supplemental Table S2. The QCMs were blinded CRMs or non-certified RMs and their results were used by JRC-IRMM to assess method trueness per laboratory at the time of the tests on the candidate CRM. Because EM methods are not typically calibrated on a daily basis, an additional QCM consisting of highly monomodal spherical polystyrene particles was provided to allow for further verification of the performance of the electron microscopes. The results of the QCMs were evaluated according to the procedure described in ERM Application Note 1 (Linsinger 2010). If the measurement result obtained on the QCM and provided by the laboratory significantly differed from the QCM’s certified or assigned reference value, then the dataset was excluded from the ERM-FD102 value assignment procedure.

The success of such a characterisation approach also depends on the measurement uncertainties that accompany the participants’ measurement results: unrealistic measurement uncertainties, in particular when underestimated, could cause a disagreement of valid data with the proposed certified value, and hence can jeopardise the certification of the candidate CRM. Therefore, all participants were asked to carefully estimate and report the uncertainties of their measurement results. The reported uncertainty value should include uncertainty contributions from all relevant and significant uncertainty sources. All participants were free to apply an approach of their choice to estimate these measurement uncertainties (*u*_meas_), e.g. as proposed in the *Guide to the expression of uncertainty in measurement* (ISO 2008a) or in the *Handbook for calculation of measurement uncertainty in environmental laboratories* (Magnusson et al. 2004).

All received datasets from the ERM-FD102 characterisation study were first checked for completeness and compliance with the predetermined conditions of the respective protocol and for their validity based on technical criteria (e.g. valid result of the QCM). Then, all technically valid datasets were grouped per measurement method and statistically analysed for outlying means (Grubbs’ test) and variances (Cochran’s test) at 99 % confidence levels. Finally, certified, indicative or additional material information values were then calculated for different measurement methods as the unweighted mean of the means of the retained data. The status of these values depends on the number of and the agreement between valid data. Compared to a non-certified RM, the strength of a CRM is its certified property value that can serve as a reference value over space and time. Such status is only justified if the CRM’s certified value and associated uncertainty have been established in a rigorous metrological manner. For assigning certified values, procedures at JRC-IRMM require, e.g. pooling of not less than six valid datasets. The detailed information regarding the participants’ applied methods, including sample preparation procedures, all measurement results and results of the statistical evaluations are available in the ERM-FD102 certification report (Kestens and Roebben 2014).

## Results and discussion

Measurement results are only comparable if they are traceable to the same reference. For dimensional measurement data (e.g. the diameter of particles), the ultimate metrological reference is the SI (International System of units) unit metre. An overview of the metrological traceability network for each of the ERM-FD102 certified values is described in the ERM-FD102 certification report (Kestens and Roebben 2014). Inextricably linked to metrological traceability are the unambiguous definition of the measurand and the uncertainty associated with the measurement result. Both metrological concepts emerged as challenging issues in the ERM-FD102 certification, as some reported datasets did not represent the requested property and some reported measurement uncertainties were unrealistically low or high.

In the following sections these two metrological challenges, i.e. the definition of the measurand and its correct interpretation, and the comprehensive estimation of measurement uncertainties, are discussed and illustrated with examples from the ERM-FD102 characterisation study.

### Definition and comparability of different size measurands

Most of the PSA methods make assumptions in their data analysis that strictly speaking only hold for perfectly spherical particles. For non-ideal spheres, even in the case of near-spherical particles, Merkus (2009) reported that the results can be significantly affected by the physical principle of the applied detection system and the applied evaluation algorithms. Our study confirms that different measurement processes can indeed provide different equivalent diameters (Table 1). By considering and using *particle diameter* as the ultimate PSA measurand, seemingly non-agreeing results may be incorrectly interpreted as being due to method inaccuracy. However, considering the intrinsic differences in measurement principles applied across the different PSA methods, and the different calculations often imposed on the raw signals by data conversion, processing and analysis algorithms to deconvolute and weigh the contributions of different particles or particle populations to the desired particle size parameter, it can be assumed that the generic measurand definition demands further refinement in order to distinguish adequately results that may or may not agree amongst different PSA methods. A complete description of the entire measurement process (incl. physical principle, technique and detection system, sample preparation, data weighting regime and statistical parameters) is needed to obtain an unambiguous specification of the size parameter that is actually measured and hence to allow a correct comparison of the associated results. As a consequence, the characterisation study of ERM-FD102 included several techniques: dynamic light scattering (DLS), scanning and transmission electron microscopy (SEM and TEM), centrifugal liquid sedimentation (CLS) with turbidity and refractive index optical detection systems, particle tracking analysis (PTA), small-angle X-ray scattering (SAXS), asymmetrical-flow field-flow fractionation (AF4) with laser light scattering (LS) and (differential) refractive index (RI) detectors and atomic force microscopy (AFM). A detailed summary of the measurement processes used in the different ILC studies, together with assigned values and uncertainties, is given in Table 1 and explained method-by-method in the following sections.

**Table 1:** Relevant features of the measurement processes underlying the measurands used for the characterisation of ERM-FD102, and associated assigned particle diameter values

Technique (-detection type)	Physical principle	Sample preparation and fractionation prior to detection	Type of particle detection	Data analysis	Type of diameter	Type of weighting	Averaging type	Assigned equivalent diameter (nm) ± expanded uncertainty (if applicable) (size range (nm) used in statistical analysis)	
								Size class A	Size class B
AF4-LS	Rate of diffusion by Brownian motion against a cross-flow	Diluted suspension, fractionated due to parabolic profile of laminar flow	Intensity of light scattered by ensemble of particles co-eluting at a given time point	AF4 theory	Sphere-equivalent hydrodynamic diameter	Scattered light intensity	Arithmetic mean^a^	22.9 (10–40)	87.5 (60–120)
AF4-RI	Rate of diffusion by Brownian motion against a cross-flow	Diluted suspension, fractionated due to parabolic profile of laminar flow	RI increment by ensemble of particles co-eluting at a given time point	AF4 theory	Sphere-equivalent hydrodynamic diameter	Mass/volume	Arithmetic mean^a^	20.4 (10–40)	ND
AFM (amplitude modulation intermittent contact)	Imaging of surface topography by scanning the surface with an oscillating cantilever	Diluted, dried on substrate	Identification of individual, discrete particles in AFM image	Measurement of height (with elimination of touching particles) with respect to flat surface following height scale calibration	Sphere-equivalent diameter	Number	Mode^a^	16.9 ± 1.8 (5–40)	80 ± 6 (30–90)
CLS-turbidity (line-start and homogeneous incremental)	Sedimentation rate	As-received suspension, fractionated through centrifugal force	Turbidity of ensemble of particles with the same sedimentation rate	Conversion of time to particle size through sedimentation time calibration (line-start incremental method) or conversion of sedimentation coefficients to particle size through Stokes’ law (homogeneous incremental method)	Sphere-equivalent Stokes diameter	Light extinction capacity	Mode^c^	23.9 ± 2.0 (10–40)	88 ± 7 (60–150)
CLS-RI (homogeneous incremental)	Sedimentation rate	As-received suspension, fractionated through centrifugal force	RI increment by ensembles of particles with the same sedimentation rate	Conversion of sedimentation coefficients to particle size through Stokes’ law	Sphere-equivalent Stokes diameter	Volume/mass	Mode^b^	18.0 ± 2.7 (5–35)	88 ± 7 (75–105)
DLS	Rate of diffusion due to Brownian motion	As-received suspension	Fluctuations of the intensity of light scattered by the ensemble of all particles in the measurement volume	Numerical deconvolution of autocorrelation function	Sphere-equivalent hydrodynamic diameter	Scattered light intensity	Arithmetic mean^c^	17.8 ± 1.5 (5–30)	88.5 ± 2.2 (30–200)
							Harmonic mean^b^	17 ± 4 (5–30)	84.8 ± 2.2 (30–200)
							Mode^b^	17.1 ± 2.4 (5–30)	84 ± 9 (30–200)
							Geometric mean^a^	16.8 (5–30)	85.2 (30–200)
							Median^a^	18.0 (5–30)	85.9 (30–200)
EM	Electron beam imaging based on transmitted (TEM) or secondary electrons (SEM)	Diluted, dried on substrate	Identification of individual, discrete particles in EM image	Image analysis of 2D projections of particles measured (with elimination of touching particles) in relation to length scale calibration	Area-equivalent circular diameter	Number	Mode^c^	18.2 ± 1.6 (10–40)	84.0 ± 2.1 (60–120)
							Median^c^	18.3 ± 1.7 (10–40)	83.3 ± 2.3 (60–120)
PTA	Rate of diffusion due to Brownian motion	Diluted suspension	Identification of individual particle trajectories in video image	Video analysis of tracked particles’ mean-square displacements and velocity in 2D through length scale and time calibration	Sphere-equivalent hydrodynamic diameter	Number	Mode^b^	ND	78 ± 5 (10–150)
							Arithmetic mean^b^	ND	82 ± 4 (10–150)
							Median^b^	ND	79.2 ± 2.2 (10–150)
SAXS	Angular distribution of X-rays scattered at surface of suspended particles	As-received suspension	Fluctuations of the intensity of X-rays scattered by the ensemble of all particles in the measurement volume	Guinier fit of high *q*-range of angular scattering pattern	Sphere-equivalent hydrodynamic diameter	Volume-squared	Arithmetic mean^a^	22.6 (NA)	ND
				Numerical deconvolution of angular scattering pattern		Volume	Arithmetic mean^c^	19.8	80.1
						Scattered X-ray intensity	Arithmetic mean^a^	21.4	81.0

*NA* not applicable

*ND* not detectable

^a^Additional material information

^b^Indicative value

^c^Certified value

#### Asymmetrical-flow field-flow fractionation (AF4)

AF4 is a technique which allows fractionation of particles (and macromolecules) ranging from 1 nm to about 10 μm (in diameter) based on their diffusion coefficients (Giddings et al. 1976). The separation results from the size-dependent position of particles in a laminar flow onto which a perpendicular cross-flow is superimposed that causes a diffusive flux in the opposite direction. The particle elution time can be determined with different online continuous-flow detectors (Bartczak et al. 2015). Such detector may be based on static or multi-angle laser light scattering (MALLS). Three different practical solutions exist to calculate particle size information from data obtained with a MALLS detector. A classical approach uses the measured intensity of the scattered light at multiple angles (Wyatt 1993). Each slice in the obtained elugram corresponds then to a curve that describes the angular dependence of the light scattered by the eluting particles. Fitting the curve with an appropriate algorithm (von der Kammer 2005) and extrapolating to zero angles gives the so-called root-mean-square (RMS) radius. The transformation of an RMS radius into a geometric radius is not straightforward as it depends on the particle geometry, the mass distribution within the particle and the chosen fitting model. A second approach for retrieving particle size information from light scattering (LS) experiments uses an external calibration and regression analysis based on the retention times of different particle size standards (Barahona et al. 2015). An essential requirement of this approach is that both the test and calibration materials must have similar physico-chemical properties to ensure similar elution behaviour. The third possible way is based on AF4 theory and consists of direct calculation (i.e. without calibration) of the particle size from the retention time of the eluted species. For known dimensions of the fractionation channel and under a constant cross-flow, the retention ratio can be determined empirically from the ratio of the measured void time and the retention time. The latter is correlated with the particle’s diffusion coefficient and subsequently, via the Stokes–Einstein equation, to the hydrodynamic diameter of the particles. The AF4 theory is well developed for particles that are dispersed in a simple matrix such as water (Giddings and Caldwell 1989; Myers 1997).

**Fig. 2:**
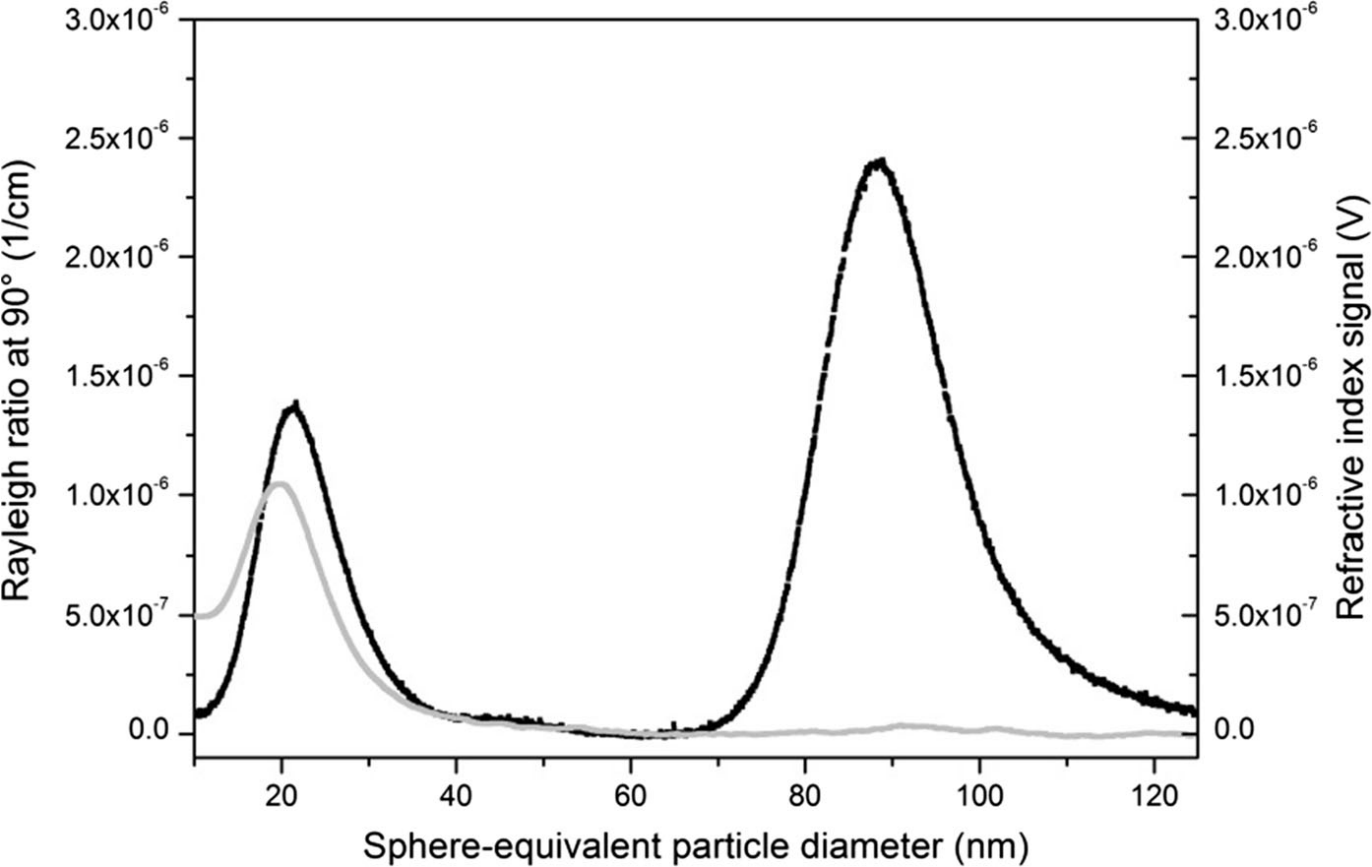
ERM-FD102 size class A and size class B particles separated (0.2 % m/v sodium dodecyl sulphate, regenerated cellulose membrane 10 kDa, 0.5 mL/min cross-flow, 1.0 mL/min elution flow) by AF4: differential refractive index (*grey curve*) and scattered light intensity (*black curve*) versus elution time (transformed into particle diameter)

In the characterisation study of ERM-FD102, only one laboratory participated with AF4 coupled to a MALLS and a (differential) refractive index (RI) detector. For the LS experiments, the laboratory used the signal from the detector that was positioned at an angle of 90°. Test samples of ERM-FD102 were diluted with purified water to a concentration of 1 g/L to avoid saturation of the detector due to the abundance of scattered light. The retention times of the void and sample peaks that were detected in the fractogram (Fig. 2), together with the experimentally determined dimensions of the separation channel, allowed the determination of the arithmetic mean particle diameter from the scattered light intensity-weighted density distribution via AF4 theory. Similarly, the retention times of the void and sample peaks of the volume-weighted fractograms (Fig. 2), as obtained from the RI detector, were used to calculate the volume-weighted arithmetic mean particle diameter. Compared to the scattered light intensity-weighted fractogram, the volume-weighted fractogram is monomodal only representing the size class A particles. Due to their larger size, the size class B particles did not influence the refractive index of the sample suspension and could hence not be detected.

#### Atomic force microscopy (AFM)

AFM generates topographical images of a surface by scanning it with a fine cantilever onto which a protruding sharp tip is attached (Binnig et al. 1986). When operated in the amplitude modulation intermittent contact mode, the measured change in amplitude triggers a feedback loop that keeps the amplitude constant by adjusting the distance between cantilever tip and surface. The feedback signal is stored for each position of the specimen and is finally used to generate an image of the specimen’s surface. Measurements of the lateral size of a nanoparticle on the scanned surface require correction of the raw data for the tip shape effect (Flater et al. 2014). However, if the particles are assumed to be near spherical and non-compressible (which is the case for ERM-FD102) and if the substrate around the particles is accessible for the tip, then the (maximum) particle height can be measured to estimate the diameter of the particles. As reported by Baalousha and Lead (2013), AFM measurements of nanoparticles can be performed under ambient air conditions and in liquid environment. While these authors demonstrated that for the given gold nanoparticles both methods can provide comparable results, it was experienced that measurements in liquid are more complicated because the particles are less firmly attached to the substrate and are hence more sensitive to perturbations.

**Fig. 3:**
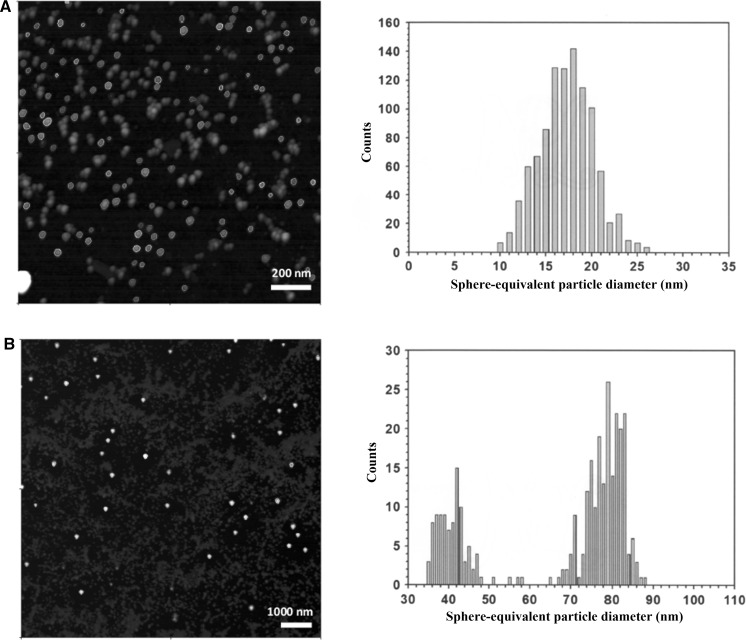
AFM images of silica nanoparticles deposited onto a silicon substrate. Non-touching particles of size class A (*top left*) and size class B (*bottom left*) were automatically detected (*coloured*) and their height measured. Graphical representations of the corresponding number-weighted PSDs are given on the right

Two laboratories participated in the ILC study with an AFM instrument that was operated in the amplitude modulation intermittent contact mode. The laboratories were allowed to follow their in-house established nanoparticle deposition procedure. To obtain regions with homogeneous distribution of predominantly single nanoparticles, the as-received material was 20–100 times diluted with purified water. 1 μL–50 μL of the diluted suspension was then brought onto pre-cleaned pieces of a silicon wafer, left to dry and imaged under ambient air conditions. Laboratories had to measure for each specimen the height of at least 1000 and 300 discrete particles of size class A and class B, respectively. Particles touching the border of the image and each other (e.g. clusters and agglomerates) had to be excluded from the PSA process. The obtained results were plotted as number-weighted particle height distributions, from which the modal values (one for size class A, one for size class B) had to be reported. Typical AFM images are shown in Fig. 3, as well as the height histograms. Due to the number ratio between size class A and size class B particles, images were acquired from scan areas of significantly different sizes. Whereas the histograms of the size class A particles were monomodal, some of the histograms of the size class B particles clearly showed to be bimodal with the second minor population corresponding to particles with a height of about 40 nm. However, as modal values were requested rather than mean values, the nominal 40 nm particle population did not affect the measurement results of the nominal 80 nm particle population. Both laboratories used fit-for-purpose artefacts with SI-traceable step-heights to calibrate the height scale, thereby providing a link between their measurement results and the SI unit metre.

#### Centrifugal liquid sedimentation (CLS)

ISO 13318-1 (2001a) classifies analytical centrifugation or CLS instruments according to their design and geometry, i.e. disc and cuvette type, and according to the type of optical detection system, i.e. Rayleigh interference or refractive index (RI) optics and turbidity. ISO 13318-2 (2007) and ISO 13318-3 (2004a) make further distinctions between instruments that are either equipped with laser light extinction or X-ray absorption detection systems. Also, instruments can be operated in the so-called line-start incremental and/or in the homogeneous incremental mode. While the homogeneous incremental method does not require calibration with a particle size standard, the line-start method can be calibrated prior to each measurement to compensate for the changing measurement parameters. The disc- and cuvette-based CLS instruments that make use of turbidity detection systems measure, as a function of time, the extinction of light from a laser (or the change in transmitted light intensity) by the particles that pass the photodetector. The measured sedimentation time is then converted into light extinction-weighted particle size information, either by means of external calibration (disc geometry) with spherical particles of known size and effective density, or via the determination of the sedimentation coefficient distribution and Stokes’ law. CLS instruments, such as analytical ultracentrifuges (AUC), which are typically equipped with RI optical detection systems measure the change in RI of the sample as the suspended particles pass the detector. Since the refractive index of the particle/medium mixture is a quantity that is related to the volume fraction of the particles in the suspension, the raw particle size results are intrinsically volume- and mass-weighted (assuming particle sphericity and homogeneous effective particle density).

**Fig. 4:**
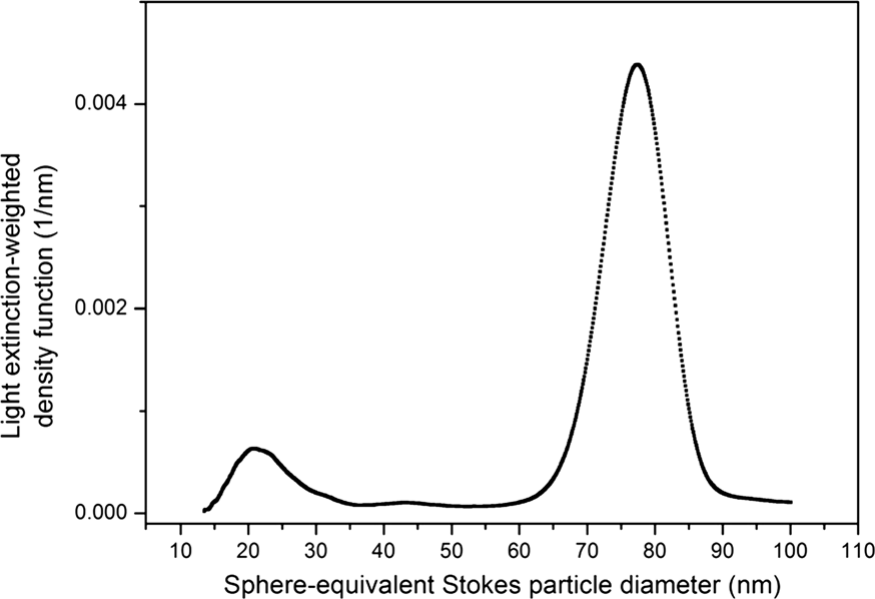
Density function of a light extinction-weighted PSD for ERM-FD102 determined using line-start CLS (disc centrifuge with turbidity optics)

For ERM-FD102, 12 laboratories were involved in the CLS ILC study. Four out of the 12 laboratories participated with homogeneous incremental CLS (2× AUC with refractive index optics and 2× instruments with turbidity optics), whereas the other eight laboratories all applied calibrated line-start (disc) methods with turbidity optical systems. Because of the low refractive index (1.460 at 546.1 nm wavelength) of bulk silica, laboratories were asked to analyse ERM-FD102 as-received, i.e. without dilution. The values to be reported were the modal Stokes values of the size class A and size class B peaks of the light extinction-weighted PSDs (turbidity) and of the mass-weighted PSDs (RI). A representative example of a light extinction-weighted PSD that was obtained by the turbidity-based CLS instruments is depicted in Fig. 4. The obtained CLS results were grouped according to the detector type, i.e. turbidity and RI. Two CLS-turbidity datasets (1× disc and 1× cuvette) were excluded from the study as the results obtained for the QCM did not agree with the QCM’s assigned value. The final group of CLS-turbidity results contained 8 valid datasets of which 7 originated from disc-based instruments. Some participants determined the modal values from density distributions belonging to a linear abscissa scale, while others used transformed density distributions having a logarithmic abscissa. According to ISO 9276-1, one can expect differences between such results, in particular for polydisperse materials covering different orders of magnitude. For ERM-FD102, the modal results of the two types of distributions agreed statistically, and therefore, no distinction was made. The disc-based instruments were all calibrated with polyvinyl chloride (PVC) particle size standards that were supplied by the company CPS Instruments Inc. (Prairieville, USA). Despite the good consistency in terms of particle size amongst the difference PVC standards, the company was unable to provide uncertainty values for the assigned particle size and effective density values. As a result, the CLS-turbidity results are only traceable to the assigned values of the PVC standards, rather than to the SI unit metre.

#### Dynamic light scattering (DLS)

DLS measures the translational diffusion coefficient of particles that randomly move in a liquid medium through Brownian motion. The diffusion coefficient is inversely related to the sphere-equivalent hydrodynamic particle diameter via the Stokes–Einstein relationship. In a first approach, known as the method of cumulants (Koppel 1972; ISO 13321 (2001b); ISO 22412 (2008b), an autocorrelation function (ACF) of the scattered light is fitted by a cumulant generating polynomial function. The first cumulant yields an average diffusion coefficient, whereas the second cumulant yields a mean-squared deviation (also known as polydispersity index) of this average. Although the cumulants method does not impose theoretically any restrictions on the shape of the distribution, in practice, the ACF is usually only fitted with a second- or third-order polynomial function, and the resulting cumulants are insufficient to determine the number of modes. Therefore, an alternative approach was followed for ERM-FD102. This approach, which is currently being considered in a next revision of ISO 22412, uses a numerical deconvolution of the ACF via Laplace transformation. The result is a scattered light intensity-weighted PSD. The main disadvantage of this Laplace transformation is that the problem is mathematically ill-posed: a given ACF can be described by an infinite number of mathematical solutions. The existing algorithms use some criteria to limit the number of possible solutions and to select the most suitable one. One such algorithm, called CONTIN, has been developed by Provencher (1982a, b). CONTIN is a generalised inverse Laplace transformation algorithm that seeks the simplest (most parsimonious) solution for a non-negative least square (NNLS) routine and is often used effectively as stand-alone PSD algorithm (Lawson and Hanson 1974). Another available algorithm, Dynals, behaves similarly to CONTIN. Since a single universally accepted Laplace transformation algorithm does not exist, most manufacturers of DLS instruments have developed their own specific algorithms that are typically based on either the CONTIN or the NNLS (or a combination of both) algorithms. Most of these algorithms differ from each other in the degree of smoothing of the ACF.

**Fig. 5:**
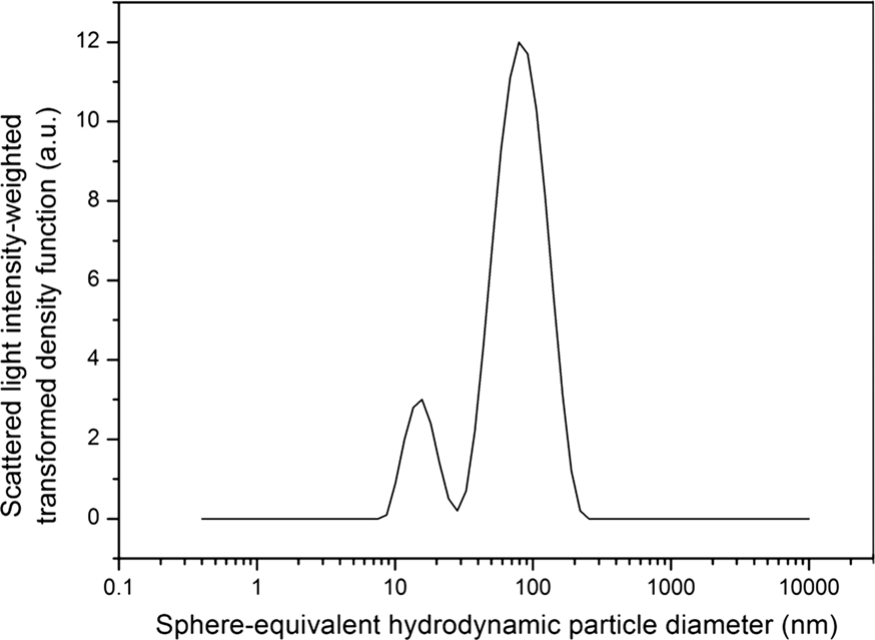
Transformed density function of a fitted scattered light intensity-weighted PSD for ERM-FD102 determined using DLS (NNLS algorithm)

A total of 18 laboratories were involved in the DLS ILC study. All laboratories were asked to apply a suitable PSD deconvolution algorithm and to report the scattered light intensity-weighted arithmetic mean diameter of the two modes (size class A and size class B). Most laboratories reported the requested parameter. However, when consulting the analysis reports that were generated by the instrument software, it was observed that there was no evident link between the reported particle size results and the exact definition of the actual measurand. For example, common instrument software presents the determined particle size results as ‘peak size’, ‘mean diameter’ or ‘mean’. The many different types of mean values that can be calculated from a PSD are described in the documentary standard ISO 9276-2 (2014) and in a paper by Finsy and De Jaeger (1991). During the ILC study, the concerned laboratories and manufacturers of commercial DLS instruments were asked to check and confirm the origin and kind of the reported results. This investigation revealed that the initially submitted datasets were a mix of different types of averages. From the DLS instrument manufacturers’ responses, it could be concluded that the majority of results were indeed calculated as arithmetic means. Nevertheless, some results appeared to be either harmonic or geometric means or even modal values. In addition to the different types of mean values, it was also observed that statistical characteristics of the PSDs (e.g. mean, modal or median values) were generally determined from transformed density functions (i.e. density function with logarithmic abscissa), whereas one instrument manufacturer uses histograms with linear abscissa. An example of a typical scattered light intensity-weighted PSD that was obtained by DLS instruments is depicted in Fig. 5. As we confirmed metrological traceability of all values corresponding to the parameters occurring in the Stokes–Einstein equation, the DLS results can be considered to be metrologically traceable to the SI unit metre.

#### Electron microscopy

Scanning and transmission electron microscopy (SEM and TEM) are used to obtain 2-dimensional (2D) projections, also of 3-dimensional objects like nanoparticles. Both SEM and TEM methods make use of a beam of primary electrons that is either stationary (TEM) or scanned over (SEM) the surface of a specimen. The backscattered or secondary electrons (SEM), or the electrons which passed through the specimen (TEM), are used to generate an electron micrograph. From such micrographs, the size and shape of individual particles can be examined through image analysis. Different geometrical particle descriptors representing different aspects of *particle size* can be derived, e.g. Feret’s and area-equivalent (circular) diameters (ISO 13322-1 (2004b); ISO 9276-6 (2008c); Rice et al. 2013; De Temmerman et al. 2012 and De Temmerman et al. 2014b).

**Fig. 6:**
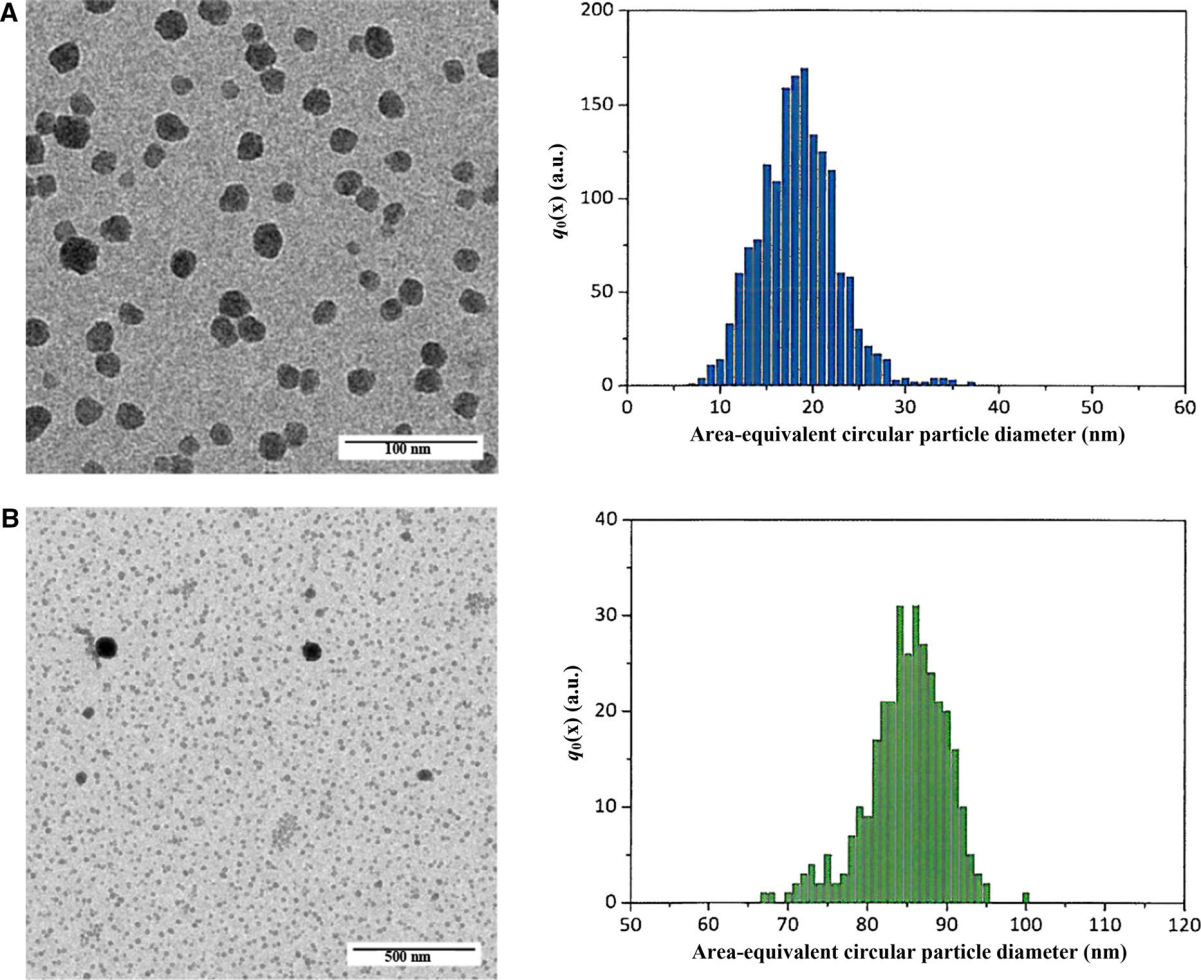
Representative examples of TEM micrographs of size class A (*top left*) and a mixture of size class A and class B particles (*bottom left*), and their corresponding histograms representing the number-weighted density distribution, *q*_0_(*x*), of area-equivalent circular particle diameters

For the characterisation of ERM-FD102, nine laboratories participated in the ILC with SEM and TEM methods. Two laboratories performed measurements using both SEM and TEM; hence, a total of 11 independent datasets were received. The measurement protocol requested the laboratories to measure for each prepared specimen the area-equivalent circular diameters of at least 1000 and 300 discrete (i.e. non-agglomerated and non-touching) particles of size class A and B, respectively. The dataset of one laboratory was excluded from the study as the size measurements were based on the length of lines bisecting the particles. The obtained results had to be plotted as number-weighted PSDs from which the associated modal values (one for size class A, one for size class B) had to be reported. Due to the much lower number of size class B particles in comparison to the number of size class A particles, laboratories were advised to apply a different magnification for each size class. However, some laboratories used a single magnification for both particle populations, thereby succeeding to determine global size distributions instead of a separate distribution for each size class. In addition to size class A and class B, several laboratories reported the presence of a third minor particle population of nominal 40 nm diameter. Since the measurement protocol instructed to only evaluate the mode and the median of the peaks of the two main particle populations, the data of the third minor population were ignored in the calculations. Although SEM and TEM have a significantly different image construction system, an unpaired two-tailed Students’ *t* test showed no significant difference (*P* < 0.05) between the mean values of the two instrument groups (SEM and TEM). Representative TEM micrographs and associated histograms of the number-weighted area-equivalent particle diameters are shown in Fig. 6. Most laboratories performed a recent calibration/verification of their instrument using artefacts with SI-traceable values, thereby linking their measurement results to the SI unit metre.

#### Particle tracking analysis (PTA)

PTA combines laser light scattering with an optical microscope equipped with a digital video camera. The camera records the speckles of light scattered by individual particles that are undergoing Brownian motion in a highly diluted suspension. The PTA software allows tracking of the individual particle trajectories based on the particle’s light scattering behaviour. The movement of the particles, which is parameterised by their mean-square displacements in 2D, allows estimation of the translational diffusion coefficient which in turn can be converted into the sphere-equivalent hydrodynamic particle diameter via a modified Stokes–Einstein equation (Filipe et al. 2010).

**Fig. 7:**
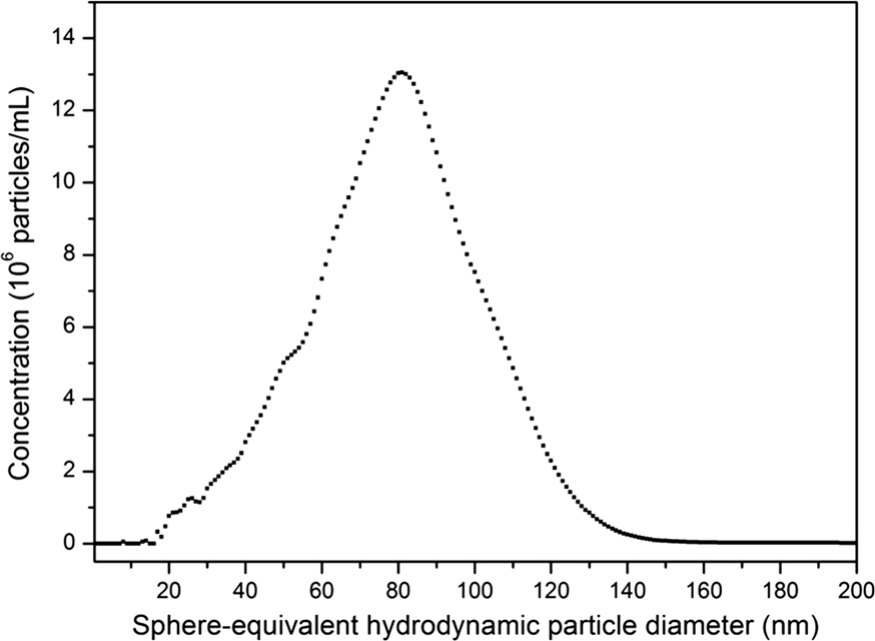
Representative PTA number-weighted PSD

Three laboratories participated in the PTA ILC studies. All laboratories were asked to report the modal, arithmetic mean and median values of the number-weighted PSDs. No specific guidelines were prescribed for sample preparation and data acquisition, allowing each of the laboratories to use their own in-house developed methodologies. Because of the relatively high particle mass fraction of ERM-FD102 (nominal 8.8 g/kg), all laboratories diluted the as-received material 1000–5000 times in purified water. In contrast to the PSDs that were obtained from the other methods, only size class B particles appeared in the number-weighted PSDs from PTA (Fig. 7). The PSD exhibits a shoulder towards lower particle sizes, which is due to the presence of the nominally 40 nm particles.

#### Small-angle X-ray scattering (SAXS)

In a SAXS experiment, a narrow beam of monochromatic X-rays is passed through a sample, i.e. a suspension of nanoparticles. The electrons of the atoms on the surface of the particles interfere with the incident X-rays thereby creating elastic scattering waves in all directions (Glatter and Kratky 1982). These scattering waves interfere with each other, forming an angular scattering pattern. The shape of the scattering curve, i.e. the angular dependence of the scattering intensity, contains information about particle size and particle shape. At small scattering vectors (*q*), the intensity only depends on the difference in electron density of the particle versus dispersing medium, concentration, particle volume and radius of gyration *R*_g_. The latter is a size parameter that corresponds to the root-mean-square (quadratic mean) distance of the atoms in the particle to the particle’s centre of mass. An overall effective mean particle size, expressed as *R*_g_ or RMS radius (similar to the RMS radius from AF4-MALLS), can be determined by fitting the high *q*-range of the scattering curve with the natural logarithm of a Gaussian function (Guinier and Fournet 1955). For spherical particles with a homogeneous density, *R*_g_ can be converted into an equivalent spherical radius by multiplying with a shape factor of 0.775. The theoretical upper limit (in diameter) to the validity of the Guinier method is about 70 nm (ISO 17867 2015b). Alternatively, the Fourier-transformed scattering curve can be numerically deconvoluted yielding not one average diameter, but a full PSD (Glatter 1977). This model fitting procedure is inherently more representative since it uses a larger portion of the scattering curve. It has recently been included in the documentary standard ISO 17867 (2015b).

Before the launch of the ILC studies, a certified or at least an indicative value was targeted for SAXS. Unfortunately, only two laboratories participated. Both laboratories received different units of the ERM-FD102 test samples. Only one laboratory returned a measurement dataset. On that basis, the results could only be reported as additional material information. The laboratory reported results from the Guinier and the model fitting methods. The scattering curve showed two linear regimes at low *q*-range corresponding with two main particle populations. A Guinier fitting of these *q*-range regimes resulted in sphere-equivalent mean diameters*,* of 22.6 nm and 51.2 nm. The silica particles of size class B could not be detected as their size was above the upper limit of detection of 70 nm. Shortly after the ILC study, the question arose whether the Guinier measurand is actually volume-squared-weighted, intensity-weighted or rather volume-weighted. This issue was presented to ISO TC24/SC4 WG10 and other SAXS experts and it was concluded that the particle size results obtained from a Guinier fit are volume-squared-weighted. The underlying rationale for the volume-squared-weighted radius is that the square of the radius of gyration, which is determined from the Guinier plot, is actually a ratio of moments of the size distribution (Glatter and Kratky 1982). The relation between the volume-squared-weighted radius and the scattered X-ray intensity- and volume-weighted radii was investigated for different sets of simulated scattering patterns of polydisperse systems by Pauw (2014). The Guinier fit was applied to the different scattering patterns and the obtained results showed that the results from the scattered X-ray intensity- and volume-weighted results over- and underestimated the expected value from the fit, respectively. The model fitting approach proved to be more informative.

**Fig. 8:**
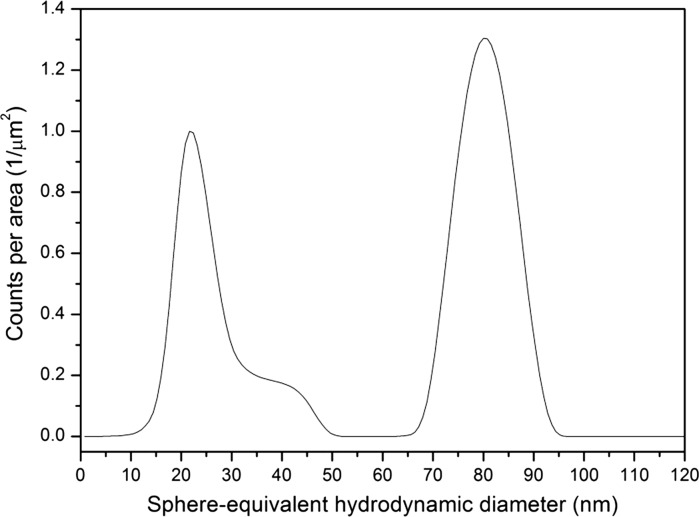
Scattered X-ray intensity-weighted PSD obtained by fitting a trimodal particle population to the indirect Fourier transformation of the SAXS scattering curve

After subjecting the raw scattering curves to indirect Fourier transformation, both size class A and size class B particle size populations could be distinguished from the volume- and scattered X-ray intensity-weighted PSDs. Also, the nominal 40 nm particles, which appeared as a shoulder to the size class A peak, were detected (Fig. 8).

#### Comparison of the values certified for different measurands

Comparing the assigned values and taking into account their associated expanded uncertainties (Table 1), it is observed that, in particular for size class B, not all assigned values agree (e.g. DLS versus PTA, DLS versus AFM/EM and EM versus PTA). For size class A, differences are less pronounced except for the CLS-turbidity results. However, the CLS results are only traceable to the assigned size values of the PVC calibrants and not to the SI (metre) as is the case for the certified values of the other methods (Kestens and Roebben 2014). The results of our study demonstrate that the term *particle size* is not sufficiently specific to define the measurand. A refinement of the measurand definition, based on a more detailed description of the entire measurement process, is essential. Furthermore, it can also be seen that the apparent agreement of some results is merely due to the relatively large uncertainties (e.g. AFM and CLS). Some of the reported measurement uncertainties (e.g. CLS) could be significantly reduced by further improving the measurement technology, including associated calibration steps. In this case, differences between results from different measurement procedures will become even more discernible. An intriguing question is whether a similar degree of equivalence will be achieved for particle size results obtained on materials whose size distributions span several orders of magnitude. For certain applications, users could accept large measurement uncertainties; in this case there is no need for further specifying the measurand definition. However, for many nanotechnology applications large measurement uncertainties can be detrimental as they increase the probability of product failure. In that respect, the ultimate goal of any measurement process is to achieve realistic measurement uncertainties that are as low as required for the purpose, so that reliable conclusions can be drawn and appropriate actions are taken.

### Measurement uncertainties

#### Measurement uncertainties estimated by individual laboratories

Procedures at JRC-IRMM require that a certified or indicative value is only assigned to a reference material property if the measurement results of all technically valid datasets agree, within their uncertainties, with the certified/indicative range. Unfortunately, underestimation of the measurement uncertainty is often a reason for non-matching results. Laboratories that participated in the ILC studies were, therefore, explicitly asked to provide measurement uncertainties that include contributions from all relevant and significant uncertainty sources. The Eurachem/CITAC guide ‘quantifying uncertainty in analytical measurement’ (Eurachem/CITAC 2012) describes two approaches for quantifying measurement uncertainties, called also top-down and bottom-up. In the top-down approach, the measurement uncertainty is estimated by combining the individual uncertainties that stem from method performance parameters such as precision (repeatability and intermediate precision) and trueness. The bottom-up approach is typically based on a measurement model that segments the different measurement uncertainty contributions according to the different stages and parameters of the measurement procedure. If both approaches are correctly applied, then the estimated measurement uncertainties should be similar. All participants followed either a top-down or bottom-up uncertainty estimation approach, or a combination of both.

**Fig. 9:**
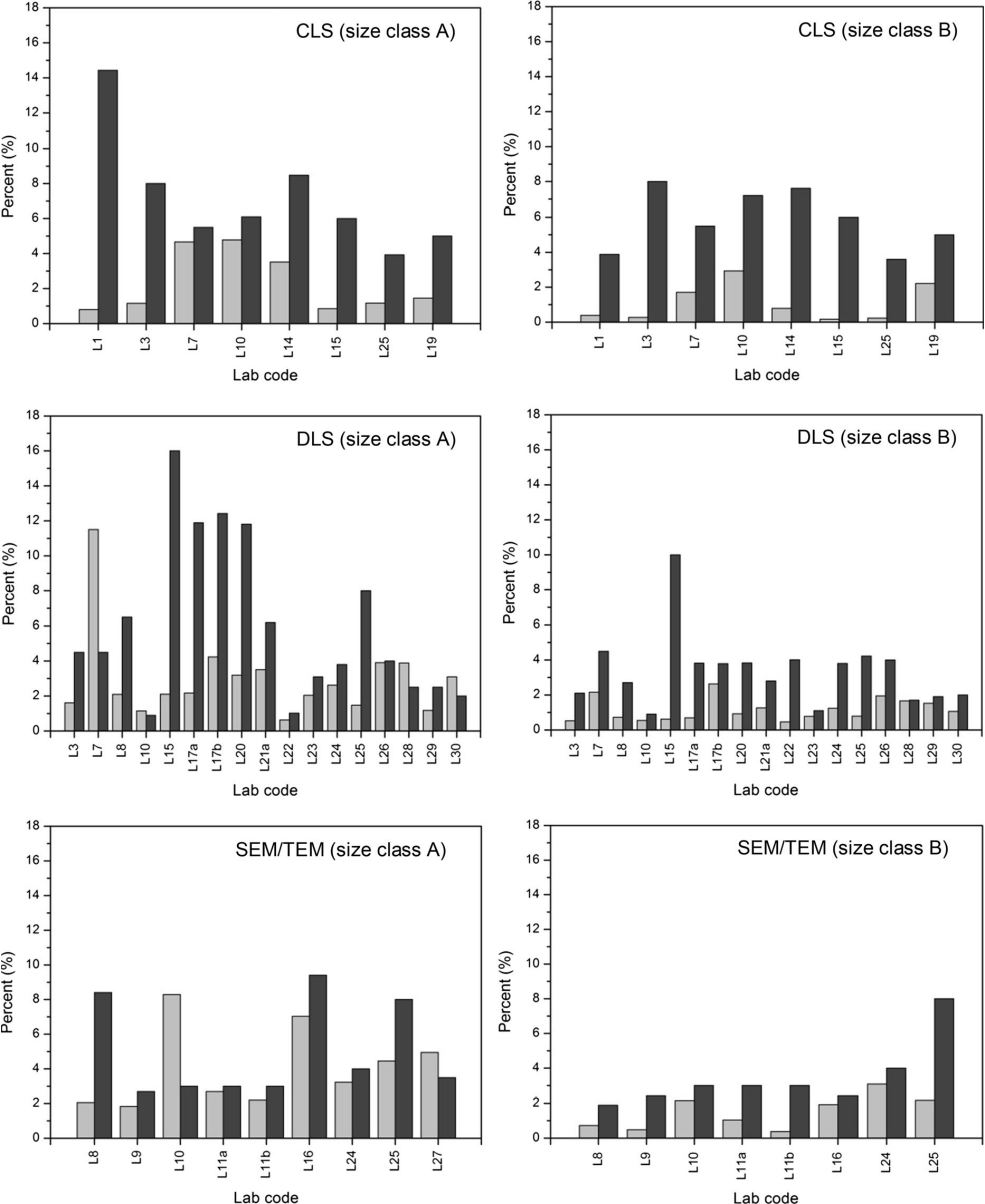
Comparison of the relative standard deviations of the measurement results (*light grey*, calculated at JRC-IRMM from the submitted data sets) with the relative standard measurement uncertainties (*dark grey*, as reported by the laboratories)

To judge whether the provided measurement uncertainties were realistic, the relative standard measurement uncertainties (for a single result) as reported by the laboratories were compared with the relative standard deviations (RSDs) that were calculated from the different aliquot results. The latter reflects the combined variation arising from method repeatability and intermediate precision (day-to-day variation). The relative standard measurement uncertainty should additionally include contributions that account for method trueness (systematic bias). As this component is not covered by the RSD, the relative standard measurement uncertainty estimated from measurements performed on a homogeneous test material should be larger than the RSD. As can be seen from Fig. 9, six laboratories reported relative standard measurement uncertainties that were smaller than the corresponding RSDs, thereby indicating that the relative standard measurement uncertainty is most likely underestimated. Depending on the approach followed when combining the results from different laboratories to determine a certified value, such underestimated measurement uncertainties could result in an unrealistic uncertainty of the certified value.

#### Uncertainties of the certified values

The uncertainties (*U*_CRM_ and *U*_CRM_CLS_) that accompany the certified and indicative values of ERM-FD102 take into account the standard uncertainty contributions from the characterisation study (*u*_char_), potential between-unit inhomogeneity (*u*_bb_) and potential degradation during transport (*u*_sts_) and long-term storage (*u*_lts_). Homogeneity and stability studies were only conducted with DLS and CLS, whereas, in theory, independent homogeneity and stability assessments are required for each measurand. Of all the methods that were included in the characterisation study, DLS and CLS are most sensitive to the presence of agglomerates/aggregates which is the main source for heterogeneity and the main indication for sample degradation. In theory also EM could be used to assess the degree of agglomeration/aggregation. However, most established image analysis routines typically apply morphology-based image processing algorithms, which omit particles whose sizes and/or shapes do not satisfy specific pre-defined criteria. Moreover, EM methods can be hampered by low statistical accuracy since only a limited number of particles can be counted and measured. As a result, EM methods are less suitable than a combination of DLS and CLS data for assessing the homogeneity and stability of near-spherical nanoparticle RMs. For that reason, the *u*_bb_, *u*_sts_ and *u*_lts_ values obtained from the DLS homogeneity and stability studies, and which were larger than those assessed by CLS, were considered and used as conservative, though still realistic contributions in the calculations of *u*_CRM_ of the other measurands (Eq. 1). The uncertainty budget of the CLS method (turbidity detection) also included 2.5 % and 2.2 % relative uncertainty contributions from the effective particle density (*u*_ρ_) and from the use of a common type of calibrants (*u*_cal_), respectively (Eq. 2). The different contributions were combined to estimate the expanded uncertainty of the certified value with a coverage factor *k* = 2 as

1 $$U_{\text{CRM}} = k \cdot \sqrt {u_{\text{char}}^{ 2} + u_{\text{bb}}^{ 2} + u_{\text{sts}}^{ 2} + u_{\text{lts}}^{ 2} \,}$$

2 $$U_{{{\text{CRM}}{\_}{\text{CLS}}}} \,\, = k \cdot \sqrt {u_{\text{char}}^{ 2} + u_{\text{bb}}^{ 2} + u_{\text{sts}}^{ 2} + u_{\text{lts}}^{ 2} \, + \,u_{\text{cal}}^{ 2} + \,u_{\rho }^{ 2} \,}.$$

**Fig. 10:**
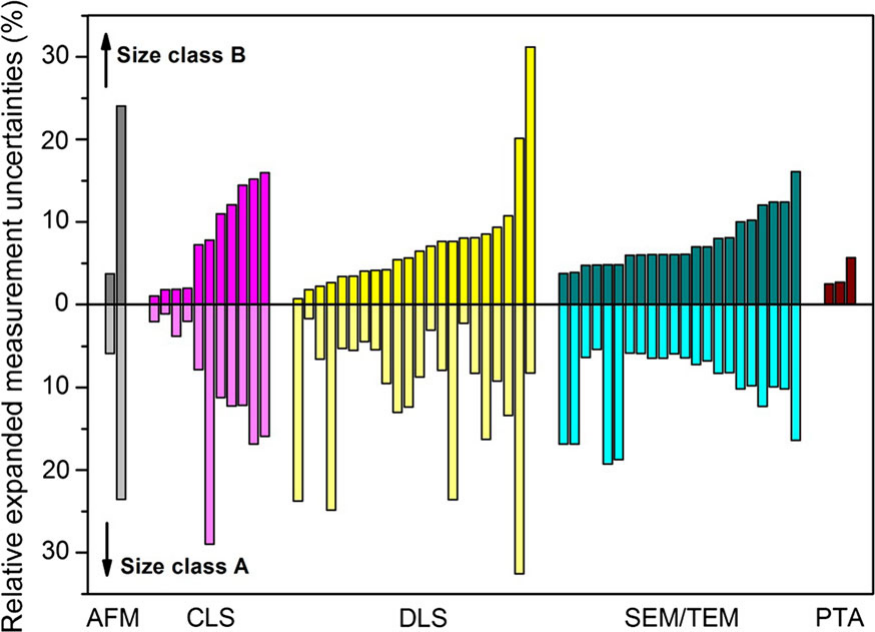
Relative expanded measurement uncertainties (*k* = 2) as reported by the participating laboratories: each *bar* corresponds to the arithmetic mean of a different dataset of ERM-FD102

Because we have shown that some laboratories had underestimated their measurement uncertainties (Fig. 9), and also because of the large differences between the uncertainties of different laboratories (Fig. 10), it was decided not to use the measurement uncertainties from the laboratories in the calculation of *u*_char_. Instead, *u*_char_ was estimated from the standard deviation (*s*) of the *n* reported laboratory mean values, according to the equation (Eq. 3) given in ISO Guide 35 (2006):

3 $$u_{\text{char}} = \,\frac{s}{\sqrt n }.$$

## Conclusions

The ERM-FD102 ILC results confirmed that the term *particle size* does not sufficiently describe the exact quantity that is measured across different PSA methods. Our findings show that the equivalent diameter values of some measurement methods do not agree and that the results of some other measurement methods only agree due to their relatively large uncertainties. For a reliable comparison of particle size, we propose a more detailed specification of the measurand ‘particle size’, including the physical principle of the measurement method and the data analysis procedures. An incomplete specification leaves room for misinterpretation along with ambiguity about the meaning or the fitness-for-purpose of the reported data.

Our study has led to the development of a new reference material with certified values and uncertainties that can be used for assessing the reliability of several particle size analysis methods. While many challenges remain, for example in establishing clear and practical pathways for metrological traceability of the measurement results, the findings of this study have shown that there is a potential to improve the understanding of measurement uncertainty in the field of nanoparticle size analysis.

## Electronic supplementary material

Below is the link to the electronic supplementary material.

Supplementary material 1 (DOCX 43 kb)

## Footnotes

1 According to ISO Guide 35 (2006), the term *characterisation* refers to the process of determining the property values of a reference material, as part of the certification process.
