# Supplementary material for: Challenges in the size analysis of a silica nanoparticle mixture as candidate certified reference material
Source: J Nanopart Res. 2016 Jun 23;18:171. doi: 10.1007/s11051-016-3474-2 (PMC4917587; doi:10.1007/s11051-016-3474-2)
Supplement: Supplementary file 1 — Supplementary material 1 (DOCX 43 kb) [file 11051_2016_3474_MOESM1_ESM.docx]

**Challenges in the size analysis of a silica nanoparticle mixture as candidate certified reference material**

**Vikram Kestens ^1^*, Gert Roebben ^1^, Jan Herrmann ^2^, Åsa Jämting ^2^, Victoria Coleman ^2^, Caterina Minelli ^3^, Charles Clifford ^3^, Pieter-Jan De Temmerman ^4^, Jan Mast ^4^, Liu Junjie ^5^, Frank Babick ^6^, Helmut Cölfen ^7^, Hendrik Emons ^1^**

^1^ Institute for Reference Materials and Measurements (IRMM), Joint Research Centre (JRC), European Commission, Retieseweg 111, B-2440 Geel, Belgium

^2^ National Measurement Institute Australia, Nanometrology Section, 36 Bradfield Road, West Lindfield, NSW 2070, Australia

^3^ Analytical Science Division, National Physical Laboratory, Hampton Road, Teddington, Middlesex, TW11 0LW, UK

^4^ Service Electron Microscopy, Veterinary and Agrochemical Research Centre (CODA-CERVA), Groeselenberg 99, B-1180 Brussels, Belgium

^5^  Division of Nanoscale Measurement and Advanced Materials, National Institute of Metrology, No. 18, Bei San Huan Dong Lu, Beijing, China

^6^ Technische Universität Dresden, Institut für Verfahrens- und Umwelttechnik, 01062 Dresden, Germany

^7^ Physical Chemistry, Department of Chemistry, University of Konstanz, Universitätsstraße 10, D-78457 Konstanz, Germany

* Correspondence: Vikram Kestens, e-mail: [vikram.kestens@ec.europa.eu](mailto:vikram.kestens@ec.europa.eu), tel.: +32 14 571 614, fax: +32 14 517 548

**Supplemental Table S1** Köstrosol 1530 and Klebosol 30R50 starting material information

| **Property** | **Specifications** | |
| --- | --- | --- |
|  | **Köstrosol 1530** | **Klebosol 30R50** |
| Batch identification | K430 | 19019/L1 |
| Appearance | Slightly turbid | Milky turbid |
| Nominal particle diameter | 20 nm | 40 nm and 80 nm |
| Specific surface area | 160-210 m^2^/g | 40-60 m^2^/g |
| Nominal SiO_2_ mass fraction | 300 g/kg | 300 g/kg |
| Free alkalinity as Na_2_O | ≤ 0.3 g/kg | ≤ 0.2 g/kg |
| pH (at 25 °C) | 9-10 | 8.5-9.5 |
| Viscosity (at 25 °C) | 6 mPa·s | n.a. ^1)^ |
| Suspension density (at 20 °C) | 1.2 g/cm^3^ | 1.2 g/cm^3^ |
| Average particle aspect ratio ^2)^ | 1.1 ^3)^ | 1.0 ^3)^ |

^1)^ Information not available from supplier

^2)^ Ratio of the major diameter (length) to the minor diameter (width) of a fitted ellipse

^3)^ Preliminary characterisation by means of TEM

**Supplemental Table S2** Summary of relevant measurement conditions as prescribed by the measurement protocol

| **Technique** | **Measurement protocol and parameters** |
| --- | --- |
| AF4-LS  AF4-RI | *Number of replicates per unit of candidate CRM (ERM-FD102):*   - 2 aliquots   *Quality control material (QCM):*   - None   *Specimen preparation strategy:*   - If for technical reasons dilution was required, then purified water (resistivity of 18.2 MΩ.cm at 25 °C), which had undergone an additional filtration process with 0.1 μm filter pore size, had to be used.   *Measurement conditions:*   - Participant’s own operating procedure   *Reporting of measurement results:*   - Scattered light intensity- and volume-weighted arithmetic mean particle diameters |
| AFM | *Number of replicates per unit of candidate CRM (ERM-FD102):*   - 2 test specimens   *Quality control material (QCM):*   - Nanosphere Size Standard 3080A ^1)^ * - Monomodal suspension of polystyrene latex nanoparticles - Assigned mean diameter: 81 nm ± 3 nm (*k* = 2) * - Mass fraction: 10 g/kg   *Specimen preparation strategy:*   - Participant’s own operating procedure   *Number of particles to be imaged and analysed per test specimen:*   - QCM: at least 1000 discrete particles - ERM-FD102: at least 1000 discrete particles of size class A and 300 discrete particles of size class B from at least 2 different randomly (but widely separated) selected scan areas   (Foreign artefacts, agglomerates and particles touching the border of the field of view had to be excluded)  *Reporting of measurement results:*   - Modal value from a number-weighted particle size (height) distribution |
| CLS (line-start) | *Number of replicates per unit of candidate CRM (ERM-FD102):*   - 2 aliquots   *Quality control material (QCM):*   - ERM-FD304 ^2)^ *   *Specimen preparation strategy:*   - It was strongly recommended to analyse the samples as-received. If for technical reasons dilution was required, then purified water (resistivity of 18.2 MΩ.cm at 25 °C), which had undergone an additional filtration process with 0.1 μm filter pore size, had to be used.   *Measurement conditions:*   - The density gradient had to be built up from mixtures of water-based low density and high density liquids (e.g., sucrose solutions, etc.). The density liquids and density gradient had to be freshly prepared on the same day of the measurement. The use of dispersant agents was not allowed. - Each measurement run had to be preceded by an independent calibration run. - For the silica particles of ERM-FD102, an effective particle density of 2.0 g/cm^3^ had to be used.   *Reporting of measurement results:*   - Modal Stokes diameter value from a light extinction -weighted particle size distribution |
| CLS (homogeneous) | *Number of replicates per unit of candidate CRM (ERM-FD102):*   - 2 aliquots   *Quality control material (QCM):*   - Nanosphere Size Standard 3080A ^1)^ * - Monomodal suspension of polystyrene latex nanoparticles - Assigned mean diameter: 81 nm ± 3 nm (*k* = 2) * - Mass fraction: 10 g/kg   *Specimen preparation strategy:*   - It was strongly recommended to analyse the samples as-received. If for technical reasons dilution was required, then purified water (resistivity of 18.2 MΩ.cm at 25 °C), which had undergone an additional filtration process with 0.1 μm filter pore size, had to be used.   *Measurement conditions:*   - The density gradient had to be built up from mixtures of water-based low density and high density liquids (e.g., sucrose solutions, etc.). The density liquids and density gradient had to be freshly prepared on the same day of the measurement. The use of dispersant agents was not allowed. - For the silica particles of ERM-FD102, an effective particle density of 2.0 g/cm^3^ and a refractive index of 1.46 had to be used.   *Reporting of measurement results:*   - Scattered light intensity- and volume-weighted modal Stokes particle diameters |
| DLS | *Number of replicates per unit of candidate CRM (ERM-FD102):*   - 3 aliquots, each aliquot had to be measured 3 times under repeatability conditions   *Quality control material (QCM):*   - ERM-FD304 ^2)^ *   *Specimen preparation strategy:*   - It was strongly recommended to analyse the samples as-received. If for technical reasons dilution was required, then purified water (resistivity of 18.2 MΩ.cm at 25 °C), which had undergone an additional filtration process with 0.1 μm filter pore size, had to be used. - Measurement cuvettes made of high quality optical glass were preferred, but disposable plastic cuvettes were allowed too.   *Measurement conditions:*   - Temperature = 25.0 °C ± 0.3 °C - Equilibration time = 300 s - Viscosity of dispersion liquid (water) = 0.8872 mPa.s (or cP) (at 25 °C) - Refractive index of dispersion liquid (water) = 1.330 (at 25 °C)   *Reporting of measurement results:*   - Scattered light intensity-weighted harmonic mean diameter was requested during the tendering stage, however, during the ILC study, this was changed to scattered intensity-weighted arithmetic mean diameter. |
| SEM/TEM | *Number of replicates per unit of candidate CRM (ERM-FD102):*   - 2 test specimens   *Quality control material (QCM):*   - ERM-FD100 ^2)^ * - Nanosphere Size Standard 3100A ^1)^ * - Monomodal suspension of polystyrene latex nanoparticles - Assigned mean diameter: 100 nm ± 3 nm (*k* = 2) * - Mass fraction: 10 g/kg   *Specimen preparation strategy:*   - Participant’s own operating procedure   *Number of particles to be imaged and analysed per test specimen:*   - QCM: at least 1000 discrete particles - ERM-FD102: at least 1000 discrete particle of size class A and 300 discrete particles of size class B from at least 2 different randomly (but widely separated) selected scan areas   (Foreign artefacts, agglomerates and particles touching the border of the field of view had to be excluded)  *Reporting of measurement results:*   - Number-weighted equivalent circular modal and median particle diameters |
| PTA | *Number of replicates per unit of candidate CRM (ERM-FD102):*   - 3 aliquots, each aliquot had to be measured 5 times under repeatability conditions   *Quality control material (QCM):*   - Nanosphere Size Standard 3080A ^1)^ * - Monomodal suspension of polystyrene latex nanoparticles - Assigned mean diameter: 81 nm ± 3 nm (*k* = 2) * - Mass fraction: 10 g/kg   *Specimen preparation strategy:*   - Test samples had to be diluted in purified water (resistivity of 18.2 MΩ.cm at 25 °C) which had undergone an additional filtration process with 0.1 μm filter pore size.   *Measurement conditions:*   - Temperature = 25.0 °C ± 0.3 °C - Equilibration time = at least 30 s - Viscosity of dispersion liquid (water) = 0.8872 mPa.s (or cP) (at 25 °C) - Refractive index of dispersion liquid (water) = 1.330 (at 25 °C)   *Reporting of measurement results:*   - Number-weighted modal, mean and median diameters |
| SAXS | *Number of replicates per unit of candidate CRM (ERM-FD102):*   - 2 aliquots   *Quality control material (QCM):*   - ERM-FD100 ^2)^ *   *Specimen preparation strategy:*   - It was strongly recommended to analyse the samples as-received. If for technical reasons dilution was required, then purified water (resistivity of 18.2 MΩ.cm at 25 °C), which had undergone an additional filtration process with 0.1 μm filter pore size, had to be used.   *Measurement conditions:*   - Participant’s own operating procedure   *Reporting of measurement results:*   - mean Guinier radius as well as the mean particle diameter (corresponding to an scattered X-ray intensity-weighted and volume-weighted size distribution) |

* Information was not made available to the participants during the ILC studies

^1)^ Thermo Scientific, brand of Thermo Fischer Scientific, Inc. (Fremont, CA, USA)

^2)^ JRC-IRMM (Geel, BE)
